# Is there a peer status gradient in mortality? Findings from a Swedish cohort born in 1953 and followed to age 67

**DOI:** 10.1093/eurpub/ckad030

**Published:** 2023-02-28

**Authors:** Ylva B Almquist, Viveca Östberg, Bitte Modin

**Affiliations:** Department of Public Health Sciences, Centre for Health Equity Studies (CHESS), Stockholm University, Stockholm, Sweden; Department of Public Health Sciences, Centre for Health Equity Studies (CHESS), Stockholm University, Stockholm, Sweden; Department of Public Health Sciences, Centre for Health Equity Studies (CHESS), Stockholm University, Stockholm, Sweden

## Abstract

**Background:**

Similar to having a less advantaged socioeconomic position, children in lower peer status positions typically experience a situation characterized by less power, influence and command over resources, followed by worse health outcomes. The aim of this study was to examine whether peer status position is further associated with increased risks for premature all-cause mortality.

**Methods:**

Data were drawn from a 1953 cohort born in Stockholm, Sweden. Peer status positions were established through survey data on peer nominations within the school class at age 13, whereas national registers were used to identify all-cause mortality across ages 14–67. Differences in hazard rates and median survival time, according to peer status position, were estimated with Cox regression and Laplace regression, respectively.

**Results:**

Although differences in hazard rates were not large, they were consistent and clear, also after taking childhood socioeconomic status into account. Regarding median survival time, the number of years lost increased gradually as peer status decreased, with a difference of almost 6 years when comparing individuals in the lowest and highest positions.

**Conclusions:**

Children’s positions in the peer status hierarchy play a role for their chances of health and survival, pointing to the relevance of addressing opportunities for positive peer interaction and mitigating any adverse consequences that may stem from negative experiences within the peer context.

## Introduction

Numerous studies have established a mortality gradient following from individuals’ positions in the societal hierarchy: having a more disadvantaged position in adulthood—whether indicated by class background, income or education—has consistently been linked to increased risks of premature all-cause and cause-specific mortality.^1–3^ Similar findings have been reported for childhood social position,^4^ also after accounting for socioeconomic circumstances in adulthood,^5^^,^^6^ thereby implying that conditions in the early stages of the life course make up a unique and persistent contribution to an individual’s chances of a long and healthy life.

Childhood is a period characterized by profound developmental change—not only in the cognitive and physical domains but also in the social realm. During this period, children’s time spent away from the parents and the family home increases.^7^ While the social position of the parents continues to set important boundaries for a child’s opportunities to develop, the importance of the peer group gradually grows and peers shift into more prominent agents of socialization.^8^ A pertinent way of expanding the inquiry into the role of early life conditions for mortality and survival is therefore to target settings outside the family, such as the peer group.

The school class constitutes one of the principal arenas for peer interaction during childhood; an arena in which the child is more or less forced to participate every weekday and for a period of several years. Although every class tends to develop their own unique pattern of social relations over time, a common consequence of these relationship-building activities is the emergence of status hierarchies where some children are ascribed a high-status position by their classmates, and others end up in the middle or at the bottom of this hierarchy. The peer status hierarchy looks exceedingly similar between boys and girls, although it should be noted that status is primarily drawn from relationships with classmates of the same gender.^9^

Children’s peer status positions can be said to reflect the extent to which they are accepted, integrated, and respected members of the group.^9^ Comparable to how one could describe issues related to occupying a disadvantaged social position in adulthood, having lower peer status typically reflects a situation characterized by less power, influence and command over resources.^9^^,^^10^ As such, peer status is more than just a proxy for familial background, past experiences, competencies, abilities or behaviours—it might also bear important consequences in and of itself. Previous studies have indeed provided evidence for a peer status gradient in a wide range of short-term and long-term health outcomes,^9^^,^^11–17^ net of childhood social position, individual child characteristics and behavioural problems.^18^^,^^19^

It nevertheless remains to be explored whether peer status position—in a similar way as for status assessed through the parents’ position in the societal hierarchy—is associated with the subsequent risk of mortality. Drawing on data from a Stockholm cohort born in 1953, the aim of this study is therefore to examine the association between children’s status position among peers in the school class (age 13) and mortality from middle childhood up until retirement age (ages 14–67). The research questions are as follow: (i) What are the differences in (a) the risk of premature all-cause mortality, and (b) median survival time, across peer status positions? (ii) Do any such differences remain after accounting for gender and socioeconomic living conditions related to the family?

## Methods

### Data material

The current study is based on The Stockholm Birth Cohort Multigenerational Study (SBC Multigen), established in 2017/18 through a probability matching between two longitudinal datasets.^20^ The first dataset was The Stockholm Metropolitan Study (SMS), defined as individuals who were born in 1953 and living in the greater Stockholm metropolitan area in 1963 (*n* = 15 117). These data were based on information from surveys as well as national and local registers. The SMS was completely de-identified in 1986. The second dataset was RELINK53 (*n* = 2 390 753), comprising all individuals born in 1953 and living in Sweden in 1960, 1963 and/or 1968 as well as their family linkages. RELINK53 consists of anonymized data compiled over a large set of national registers, linked by personal identification numbers at the individual level. By using an algorithm based on variables identical to both datasets, 14 608 of the original SMS cohort members could be positively matched and thereby included in the SBC Multigen. The Swedish Ethical Review Authority has approved the creation of the SBC Multigen as well as the research proposed in the current study (no. 2017/34-31/5; 2017/684-32).

### Variables

Premature all-cause mortality was defined as death from any cause occurring between 1 January 1967 and 31 December 2020 (ages 14–67), as recorded in The Swedish Cause of Death Register.

Cohort members attending schools in the greater Stockholm area were invited to partake in the School Study of 1966. For school classes in 6th grade (age 13), all students (whether part of the cohort or not) were asked to answer a set of so-called sociometric questions.^18^^,^^21^ Peer status was established by the question ‘Whom in this class do you best like to work with at school?’. Students were instructed to nominate three classmates in no particular order. The number of received nominations could subsequently be calculated for each cohort member. For the purposes of the current study, and in line with categorizations used in previous studies,^12^^,^^22^ four peer status positions were constructed: marginalized (0 nominations), low status (1 nomination), intermediate status (2–3 nominations) and high status (4 or more nominations).

Control variables were gender, school class size (age 13), household educational level (age 7), household occupational class (age 10), family type (age 10) and household receipt of social welfare benefits (ages 0–12). The rationale for including gender is that men tend to have higher risks for premature mortality compared to women^23^ and that sociometric nominations mainly are given by persons of the same gender.^9^ While not observed empirically in past studies,^9^ school class size can theoretically influence the distribution of individuals into peer status categories. The remaining control variables are all considered as reflective of childhood socioeconomic living conditions and have, as such, been linked to both peer status^24^ and premature mortality.^6^^,^^25^

An overview of the variables, including an account of which registers the information was drawn from, is presented in Supplementary table S1.

### Study sample

Cohort members were excluded from the analysis if they: (i) had died between 1 January 1964 and 31 December 1966 (*n* = 7); (ii) did not attend 6th grade in 1966 (*n* = 2220); (iii) attended a school class consisting of <10 students (*n* = 260); and (iv) lacked information about peer status position (*n* = 12). None of the control variables contributed with any further exclusions. Reasons for excluding individuals attending school classes in other grades or school classes consisting of <10 students were that the sociometric test was only performed for classes in 6th grade, and that the peer status distribution tends to be rather truncated in very small classes. Implementation of the above-specified exclusion criteria resulted in an analytical sample of 12 109 individuals (83%).

### Statistical analysis

The association between peer status position and all-cause mortality was analysed with two different, but complementary, regression-based methods. The first was Cox regression analysis, which made it possible to estimate differences in the risk of mortality (expressed as hazard ratios; HRs) by peer status position while taking time under risk into account.^26^ Age was chosen as the underlying time scale. More information about the time variables used in the Cox regression analysis is shown in Supplementary table S2. No problems with tied failure times were identified. The assumption of proportional hazards was confirmed for the model as a whole (Schoenfeld’s global test: *P *>* *0.05) as well as for all the separate variables included in the analysis.

The second method was Laplace regression analysis, which can be seen as a complement to the unitless measures derived from Cox regression. Under the assumption that the error term follows the asymmetric Laplace distribution, differences in survival time (expressed in years) at each given survival percentile can be estimated as a linear function of the independent variables included in the model.^27^^,^^28^ As such, Laplace regression allows for an interpretation in terms of time gained or lost. The results reported in the current study are based on survival time at the 50th (median) percentile and 500 bootstrap replications. Although a large proportion of the sample was censored (due to the follow-up ending) before the estimated percentile, it should be noted that estimations at lower percentiles (e.g. 10th) yielded similar results.

For the Cox and Laplace regression analyses alike, two models were estimated with a stepwise inclusion of control variables. Model 1 was adjusted for gender and school class size, whereas Model 2 additionally included socioeconomic living conditions in the family (household educational level, household occupational class, family type and household receipt of social welfare benefits). It should be noted that only low to moderate correlations were found between the indicators of socioeconomic living conditions in the family. Additional analyses found no evidence of a statistical interaction between peer status position and gender (*P *>* *0.05). Data management and analyses were performed with Stata, version 16.1.

## Results

In terms of descriptive statistics (table 1), the sample was evenly distributed in terms of gender. Across ages 14–67, a total of 11.5% of the individuals in the analytical sample had died. Around one-third had a high peer status position in childhood, a little more than one-third had an intermediate peer status position, one-fifth had a low peer status position, and about one-tenth were marginalized. School class size ranges between 10 and 40 students, with a mean and median of 26 and 27, respectively. In their childhood, during the 1960s, the vast majority of the cohort members lived in a two-parent household. Overall, most individuals had low-educated parents, a slightly lower percentage had a working-class background as compared to a middle or upper middle-class background, and ∼16% grew up in a household that received social welfare benefits. Premature all-cause mortality was more common among men and those in lower peer status positions, as well as among those with less advantaged socioeconomic backgrounds.

**Table 1:** Descriptive statistics of the study variables (*n *=* *12 109)

	*n*	%	All-cause mortality	
			%	*P*-value^a^
**All-cause mortality (ages 14–67)**				
No	10 717	88.5	–	–
Yes	1392	11.5	–	–
**Peer status position (age 13)**				<0.001
High status	3771	31.1	9.0	
Intermediate status	4519	37.3	11.2	
Low status	2460	20.3	13.6	
Marginalized	1359	11.2	15.5	
**Gender**				<0.001
Woman	6115	50.5	9.0	
Man	5994	49.5	14.0	
**School class size (age 13)**	Min = 10, Max = 40, Mean = 26; Median = 27			
**Household educational level (age 7)**				<0.001
At least one parent graduated from upper sec. school	3191	26.4	9.2	
No parent graduated from upper sec. school	8268	68.3	12.5	
Missing information	650	5.4	10.2	
**Household occupational class (age 10)**				0.001
Middle and upper middle class	6216	51.3	10.4	
Working class	5557	45.9	12.6	
Unclassified	336	2.8	12.2	
**Family type (age 10)**				0.043
Two-parent household	11 024	91.0	11.3	
Other	1085	9.0	13.4	
**Household receipt of social welfare benefits (ages 0–12)**				<0.001
No	10 231	84.5	10.6	
Yes	1878	15.5	16.2	

a Based on χ^2^ tests.

**Figure 1:**
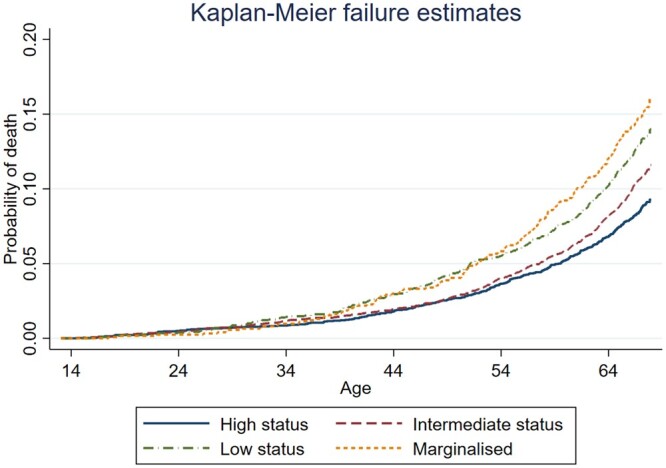
Kaplan–Meier curves with failure function: all-cause mortality (ages 14–67) (*n *=* *12 109).

Kaplan–Meier failure curves for all-cause mortality by peer status position are shown in figure 1. As illustrated, probabilities of death are fairly similar for all categories of peer status until around age 40, when the groups start diverging. From that age and through the following decade of life, the failure curves for individuals with low status and those in a marginalized position go largely in parallel. The same applied to individuals with high and intermediate status. Beyond age 60, there is a clear gradient in the probability of death across all four categories of peer status position.

Table 2 demonstrates the associations between peer status position (age 13) and all-cause mortality (ages 14–67), based on the Cox regression analyses. In Model 1, which is adjusted for gender and school class size, a clear gradient in mortality by peer status position can be seen (test for trend: *P *<* *0.05). In comparison to individuals with high status, the hazard rate for those with intermediate status and low status is 27% [HR: 1.27; 95% confidence interval (CI): 1.11, 1.45] and 69% (HR: 1.69; 95% CI: 1.37, 1.85) higher, respectively, whereas individuals in a marginalized position have a 75% higher hazard rate (HR: 1.75; 95% CI: 1.47, 2.07). While further adjustment for socioeconomic living conditions related to the family (Model 2) attenuates the HRs, the overall conclusion does not change.

**Table 2:** Associations between peer status position (age 13) and all-cause mortality (ages 14–67)

	All-cause mortality (ages 14–67)			
	Model 1^a^		Model 2^b^	
	HR	95% CI	HR	95% CI
**Peer status position (age 13)**				
High status (reference)	1		1	
Intermediate status	1.27	1.11, 1.45	1.23	1.07, 1.41
Low status	1.69	1.37, 1.85	1.52	1.31, 1.77
Marginalized	1.75	1.47, 2.07	1.63	1.37, 1.94

Results from Cox regression analysis (*n *=* *12 109), presented as hazard ratios with 95% confidence intervals.

CI, confidence interval; HR, hazard ratio.

a Adjusted for gender and school class size.

b Adjusted for gender, school class size, household educational level, household occupational class, family type and household receipt of social welfare benefits.

In table 3, the results from the Laplace regression analyses are shown. According to Model 1, which is adjusted for gender and school class size, those in a marginalized peer status position have a median survival time that is 6.73 years shorter (95% CI: −8.86, −4.61) compared to those with high status. The corresponding loss of years for those with intermediate status and low status is 2.96 (95% CI: −4.61, −1.31) and 5.61 (95% CI: −7.47, −3.75), respectively. Adjustment for family socioeconomic living conditions (Model 2) reduces the differences in median survival time across peer status positions. However, the general pattern remains clear.

**Table 3:** Associations between peer status position (age 13) and all-cause mortality (ages 14–67)

	All-cause mortality (ages 14–67)			
	Model 1^a^		Model 2^b^	
	Diff. in years	95% CI	Diff. in years	95% CI
**Peer status position (age 13)**				
High status (reference)	0		0	
Intermediate status	−2.96	−4.61, −1.31	−2.56	−4.19, −0.92
Low status	−5.61	−7.47, −3.75	−5.07	−6.93, −3.21
Marginalized	−6.73	−8.86, −4.61	−5.90	−8.02, −3.79

Results from Laplace regression analysis (*n *=* *12 109), presented as differences in median survival time (years) with 95% confidence intervals.

CI, confidence interval; HR, hazard ratio.

a Adjusted for gender and school class size.

b Adjusted for gender, school class size, household educational level, household occupational class, family type and household receipt of social welfare benefits.

## Discussion

The aim of the current study was to examine the association between children’s status position among peers in the school class and mortality from middle childhood up until retirement age. The results suggest that individuals who held lower peer status positions in childhood have increased risk of dying prematurely. While differences in hazard rates are not large, they are consistent and clear, also after accounting for socioeconomic living conditions related to the family. The patterning of median survival time is equally consistent: the number of years lost increases gradually as peer status decreases, with a difference of almost 6 years when comparing individuals in marginalized positions to those with high status. In the Swedish context, this is on par with the current educational gap in life expectancy at age 30.^29^

While no past research has specifically looked into the association between peer status position and premature mortality, the findings are consistent with inquiries into mortality risks among individuals at the very bottom of the peer status hierarchy, i.e. those who are isolated, marginalized or rejected.^30^ The results are also in line with previous findings, much of which is based on the same cohort as the present study, regarding peer status differences for a wide range of morbidity outcomes, such as affective disorders, substance misuse, suicide attempts, circulatory diseases and diabetes.^11^^,^^15^^,^^17^ It should be noted that while the outcome in the present study is all-cause premature mortality, deaths are primarily due to external causes (e.g. accidents, injuries and suicides), mental and behavioural disorders (e.g. alcohol and drug misuse) and other non-communicable diseases (e.g. cancers and cardiovascular diseases), which suggests that the same pathways—such as the material, psychosocial and health behavioural pathways^18^—linking peer status position to morbidity might also be at play here.

It is also known from previous studies that lower peer status is associated with higher risks of disadvantaged socioeconomic outcomes across the life course,^19^^,^^24^ as well as an increased likelihood of ending up in situations characterized by strong clustering of adversities in adulthood.^31^ Considering the well-established socioeconomic gradient in mortality,^2^^,^^32^ this too may provide an explanation for findings of the current study. That said, it is important to consider early antecedents to the development of both morbidity and socioeconomic conditions as they are likely to also be intertwined with the establishment of peer status positions.^33^ For example, there is an array of externalizing and internalizing problems that might lead to, as well as be caused by, low peer status^34^^,^^35^ and then further feed into poor health, disadvantaged socioeconomic conditions, and, subsequently, risks of premature death. In a similar vein, various competences, abilities and achievements could influence and be influenced by high peer status,^36^ which in turn might lead to a more positive developmental trajectory across the life course.

### Strengths and limitations

This study had a number of important strengths, but also some limitations that should be addressed. The analyses are based on a sample from the general population that was followed from childhood up until retirement age. Prospective study designs with an extensive follow-up are generally rare in the context of peer status research, which makes the current research rather unique. Concerning peer status positions, they were determined by sociometric information which provides a more objective assessment. It also avoids problems associated with direct questions about, for example, perceived popularity (which may additionally reflect a greater propensity of health risk behaviours).^37^ Though the sociometric information was only collected at one point in time, thus providing what might best be described as a snap-shot of the individuals’ peer status positions, previous research has found peer status positions to be rather stable.^38^ Another potential limitation refers to the usage of information on preferred work partner. This question captures task-specific aspects to a larger extent than e.g. questions about whom children like the best. Yet, it has been found to reflect choices based on general likability of the peers as well as an evaluation of both their social and cognitive skills.^21^

For all-cause mortality, The Swedish Cause of Death Register is a reliable source of information.^39^ The register captures all occurring deaths and, in a vast majority of cases, the exact date of death. While it would have been interesting also to examine cause-specific mortality as way of approaching the mechanisms behind mortality differences across peer status positions, the cohort is still too young, and the number of deaths too small, to enable such an analysis. Additionally, as the first study to investigate the link between childhood peer status and mortality up until retirement age, the aim was exploratory and did not set out to empirically examine possible explanations. This is nevertheless a relevant task for future studies.

Future research might also need to (re)consider the issue of confounding. Control variables in the current investigation included several indicators of socioeconomic living conditions in the family. While they offer a representative picture of the 1953 cohort’s childhood, much has happened in Swedish society since then. Educational expansion, shifts in the occupational class structure, and increased divorce rates may potentially have led to changes also in the contribution of socioeconomic living conditions for the association between peer status and mortality.

## Conclusions

This study shows that individuals in lower peer status position have increased risks of dying prematurely, amounting to a significant loss of years lived. But what can findings in this 1953 birth cohort tell us about differences in the chances of survival in children of today? The grounds for peer status might have shifted since the 1960s, when this cohort attended primary school, and there is generally a stronger awareness about the relevance of children’s peer relations for their health and wellbeing. However, there are no reasons to believe that status struggles have grown less evident or important in children’s lives, thus suggesting the long-lasting imprint that a child’s peer status position leaves can be assumed to be as profound today as compared to more than a half-century ago.

As previously discussed, peer status hierarchies are established by a complex interplay between individuals, developing over time, and bound to a given context. If the ambition, in the long run, is to mitigate the mortality differences found in this study, children need to be presented with the opportunities for positive peer interaction—no matter the competencies, abilities or behaviours they bring to the table—and supported in their endeavour to become integrated, accepted, and respected members of the peer group. While there are plenty of school-based interventions aiming to prevent bullying and loneliness in children,^40^ such programmes could be expanded to more broadly target peer relationships and the overall hierarchy of the peer group.

## Supplementary Material

ckad030_Supplementary_Data

## Data Availability

Owing to ethical regulations regarding the Stockholm Birth Cohort Multigenerational Study (SBC Multigen), access to the data is restricted. If there is interest in the unpublished data from this research article, a request can be made to the main author, who will forward it to the steering committee of the SBC Multigen.
