# Supplementary material for: Is there a peer status gradient in mortality? Findings from a Swedish cohort born in 1953 and followed to age 67
Source: Eur J Public Health. 2023 Feb 28;33(2):184–9. doi: 10.1093/eurpub/ckad030 (PMC10066469; doi:10.1093/eurpub/ckad030)
Supplement: ckad030_Supplementary_Data [file ckad030_supplementary_data.docx]

Supplementary Table S1. Study variables and sources of information.

| Study variables | Year(s) | Age(s) | Categorisation | Sources of information | Additional comment |
| --- | --- | --- | --- | --- | --- |
| All-cause mortality | 1967-2020 | 14-67 | No; Yes | The Swedish Cause of Death Register | Refers to death from any cause. |
| Peer status | 1966 | 13 | High status; Intermediate status; Low status; Marginalised | The School Study | Based on the number of received peer nominations for “Whom in this class do you best like to work with at school?”. |
| Gender | 1953 | 0 | Woman; Man | The Total Population Register |  |
| School class size | 1966 | 13 | - | The School Study | Based on the number of students in the class, including students that were not cohort members. |
| Household educational level | 1960 | 10 | At least one parent graduated from upper sec. school; No parent graduated from upper secondary school | The 1960 Census | The information refers to adults living in the cohort member’s household. In a vast majority of cases, there are two adults (of which both are the parents) whereas, in a few cases, it could also be other adult relatives/family members. |
| Household occupational class | 1963 | 10 | Middle and upper middle class; Working class; Unclassified | Occupational Registers | Occupational class refers to the head of the household (most often the cohort member’s father) |
| Family type | 1963 | 10 | Two-parent household; Other | Income Registers | ‘Two-parent household’ in a majority of cases reflect cohort members living with both biological parents and in some cases with the father/mother and the spouse. ‘Other’ refers to any other type of family constellation (including a small proportion, <1%, of missing data). |
| Household receipt of social welfare benefits | 1963 | 0-12 | No; Yes | The Social Register | The Social Register was kept at the municipal level. |

Supplementary Table S2. Time variables used in the Cox regression analysis.

| Time variables | Date | Additional information |
| --- | --- | --- |
| Origin | Date of birth | Exact information on year (1953 for all cohort members) and month was used. Day of birth was set to the middle of the month (i.e. the 15^th^). For a small proportion (less than 1%) of the individuals, only information on year was available – for those, the date was set to the middle of the year (i.e. June). |
| Entry | 1 January 1967 |  |
| Failure | Date of death | Exact information on year, month, and day of death was used. For a small proportion (less than 1%) of the individuals, only information on year or year and month was available – for those, the date was set to the middle of the year (i.e. June) and/or month (i.e. the 15^th^). |
| Exit | Date of death or 21 December 2020 |  |
